# Fabrication of novel elastosomes for boosting the transdermal delivery of diacerein: statistical optimization, *ex-vivo* permeation, *in-vivo* skin deposition and pharmacokinetic assessment compared to oral formulation

**DOI:** 10.1080/10717544.2018.1451572

**Published:** 2018-03-20

**Authors:** Diana E. Aziz, Aly A. Abdelbary, Abdelhalim I. Elassasy

**Affiliations:** a Department of Pharmaceutics and Industrial Pharmacy, Faculty of Pharmacy, Cairo University, Cairo, Egypt;; b Department of Pharmaceutics and Industrial Pharmacy, Faculty of Pharmacy, October 6 University, Giza, Egypt

**Keywords:** Diacerein, elastosomes, edge activators, transdermal delivery, *in-vivo* pharmacokinetic study

## Abstract

Diacerein (DCN) is a hydrophobic osteoarthritis (OA) drug with short half-life and low oral bioavailability. Furthermore, DCN oral administration is associated with diarrhea which represents obstacle against its oral use. Hence, this article aimed at developing elastosomes (edge activator (EA)-based vesicular nanocarriers) as a novel transdermal system for delivering DCN efficiently and avoiding its oral problems. For achieving this goal, elastosomes were prepared according to 4^1^.2^1^ full factorial design using different EAs in varying amounts. The prepared formulae were characterized regarding their entrapment efficiency percentage (EE%), particle size (PS), polydispersity index (PDI), zeta potential (ZP) and deformability index (DI). Desirability function was employed using Design-Expert^®^ software to select the optimal elastosomes (E1) which showed EE% of 96.25 ± 2.19%, PS of 506.35 ± 44.61 nm, PDI of 0.46 ± 0.09, ZP of −38.65 ± 0.91 mV, and DI of 12.74 ± 2.63 g. In addition, E1 was compared to DCN-loaded bilosomes and both vesicles exhibited superior skin permeation potential and retention capacity compared to drug suspension. *In-vivo* histopathological study was performed which ensured the safety of E1 for topical application. Furthermore, the pharmacokinetic study conducted in albino rabbits demonstrated that there was no significant difference in the rate and extent of DCN absorption from topically applied E1 compared to oral suspension. Multiple level C *in-vitro in-vivo* correlation showed good correlation between *in-vitro* release and *in-vivo* drug performance for E1 and DCN oral suspension. Overall, results confirmed the admirable potential of E1 to be utilized as novel carrier for transdermal delivery of DCN and bypassing its oral side effects.

## Introduction

Diacerein (DCN) is a structural modifying osteoarthritis drug (SMOAD) which selectively inhibits interleukins-1β (IL-1β) in human monocytes that has a significant role in cartilage degeneration (Verbruggen, 2006; El-Laithy et al., 2015). Hence, it manages the symptoms of osteoarthritis (OA), targets the underlying pathology, and arrests the disease progression (Medhi et al., 2007; Dhaneshwar et al., 2013). Being a BCS class II drug, DCN is slightly soluble in water (3.197 mg/L) with low oral bioavailability (35%–56%) (Elsayed et al., 2014). After DCN oral administration, the remaining unabsorbed drug induces laxative effect by stimulating prostaglandins (PGs) and acetylcholine release and consequently causes diarrhea (El-Laithy et al., 2015). Furthermore, DCN is orally administered frequently due to its short half-life (4 h; Moghddam et al., 2016). These factors collectively cause poor patient compliance and decreased adherence to DCN oral regimen.

Transdermal drug delivery system (TDDS) is a noninvasive, painless route that can avoid the gastrointestinal side effects associated with orally administered drugs (Moghddam et al., 2016). TDDS is designed for treatment of chronic disorders like OA (Ahad et al., 2013) as it gives consistent systemic drug levels by delivering it at a controlled and predetermined rate which reduces the dosing frequency with consequent lower side effects and improved bioavailability (Bhowmik et al., 2010). As a result, TDDS is expected to be a promising alternative mode for the delivery of DCN with the aim of improving its effectiveness and avoiding its oral side effects.

However, the absorption of most drugs across the skin is limited by its impervious anatomic barrier; stratum corneum (SC). Utilization of especially designed elastic vesicles of various compositions (e.g. ethosomes and bilosomes) is one of the proposals that had been assessed to mitigate this barrier (Touitou et al., 2000; Al-Mahallawi et al., 2015). These elastic vesicles serve as penetration enhancers that can structurally modify SC by their constituting lipids and deeply penetrate through the skin with minimal risk of vesicular wall rupture compared to conventional niosomes and liposomes (Muzzalupo et al., 2011; Kakkar & Kaur, 2013). The results of our previous work confirmed the ability of bilosomes to be used as vesicular carriers for enhancing the transdermal drug delivery (Al-Mahallawi et al., 2015; Aziz et al., 2018). In this article, bilosomes was selected as a nucleus for developing novel elastosomes by modulating bilosomal composition using various types of edge activators (EAs). Being surfactants, EAs can lower the surface tension of vesicular lipid bilayer and consequently increase their deformability (Duangjit et al., 2013). Accordingly, elastosomes were hypothesized to be more deformable than bilosomes to squeeze through skin pores to deeper tissues followed by systemic uptake via dermal blood vessels and consequently bypass the gastrointestinal side effects associated with oral route (El Zaafarany et al., 2010).

Thus, the work in this study was divided into three main goals; the first one was developing DCN elastosomes according to a 4^1^.2^1^ full factorial design using Design-Expert^®^ software to study the effect of formulation variables on their characteristics and to select the optimal formula based on the desirability function. Second, to ascertain the hypothesized enhanced skin permeation potential and retention capacity of elastosomes, the optimal elastosomes formulation was subjected to comparative *ex-vivo* permeation and *in-vivo* skin deposition studies with DCN-loaded bilosomes and drug suspension. Furthermore, histopathological study was performed using male Wistar rats to ensure the safety of the prepared elastosomes. Third, for the purpose of bioavailability assessment, a pharmacokinetic study was performed using male albino rabbits for the optimal elastosomes and the results were compared with that of orally administered DCN suspension. In addition, multiple level C *in-vitro in-vivo* correlation (IVIVC) between the resultant *in-vitro* and *in-vivo* data of both the optimal elastosomes and DCN oral suspension was also investigated.

## Materials and methods

### Materials

DCN was a kind gift from EVA Pharmaceutical Industries (Cairo, Egypt). Cholesterol (CH), Span 60 (sorbitan monostearate), Brij S2 (polyoxyethylene (2) stearyl ether), Brij C10 (polyoxyethylene (10) cetyl ether), and acetonitrile (HPLC grade) were purchased from Sigma Chemical Co. (St. Louis, MO). Sodium taurocholate (STC), Cremophor EL, and Cremophor RH 40 were acquired from BASF Co. (Florham Park, NJ). Methanol, chloroform, disodium hydrogen phosphate, potassium dihydrogen phosphate, and sodium chloride were purchased from El-Nasr Pharmaceutical Chemicals Co. (Abu-Zaabal, Cairo, Egypt). All other reagents were of analytical grade and were used as received.

### Preparation of DCN-loaded nanovesicles

DCN elastosomes were prepared using film hydration technique (Dai et al., 2013). Briefly, DCN (25 mg), 150 mg of vesicles forming materials (Span 60 and CH) in ratio 5:1 and 15 mg of STC together with different amounts of the used EA (Brij S2 or Brij C10 or Cremophor EL or Cremophor RH 40) were accurately weighted into 250 mL long-necked round-bottom flask and dissolved in 10 mL of chloroform–methanol mixture (7:3). The obtained clear organic solution was reduced to thin lipid film by slow evaporation at 60 °C under reduced pressure using a rotary evaporator (Rotavapor, Heidolph VV 2000, Burladingen, Germany) for 30 min at 90 rpm. The dry film was then hydrated using 10 mL of ultra-pure distilled water by rotating the flask in a water bath maintained at 60 °C for 30 min at 150 rpm using the same apparatus under normal pressure to form milky dispersion of DCN elastosomes. For particle size (PS) reduction, the obtained suspension was then sonicated for 30 min in a bath sonicator (Ultrasonic bath sonicator, Model SH 150-41; MTI Corporation, Richmond, CA) at 25 °C. For the purpose of comparison, DCN-loaded bilosomes (composed of Span 60, CH, and STC) were prepared using the same amounts by the previously mentioned manner. Finally, the prepared formulae were left to equilibrate overnight at 4 °C for further characterization.

### *In-vitro* characterization of DCN elastosomes

#### Determination of DCN entrapment efficiencypercentage (EE%)

EE% of DCN was estimated indirectly by measuring the amount of free (unentrapped) DCN in dispersion media (Abdelbary & AbouGhaly, 2015). The free DCN was separated from the prepared nanovesicles by centrifugation of 1 mL of the vesicular suspension at 25,000 rpm for 1 h at 4 °C using a cooling centrifuge (Sigma 3-30 KS, Sigma Laborzentrifugen GmbH, Osterode am Harz, Germany). The resultant supernatant was separated, properly diluted, and analyzed for free DCN concentration spectrophotometrically (Shimadzu, model UV-1601 PC, Shimadzu Corp., Kyoto, Japan) by measuring the ultraviolet (UV) absorbance at *λ*_max_ 258 nm. Each result was the mean of three determinations ± *SD*. Drug EE% was determined according to the following equation:

(1) $$\text{EE\%}\;\text{=}\;\left[\frac{\text{(total}\;\text{amount}\;\text{of}\;\text{DCN}\;-\;\text{unentrapped}\;\text{DCN)}}{\text{total}\;\text{amount}\;\text{of}\;\text{DCN}}\right]\;\text{×}\;\text{100}$$

#### Determination of particle size (PS), polydispersity index (PDI), and zeta potential (ZP)

The average PS, PDI, and ZP of the prepared vesicles were determined by dynamic light scattering technique using Zetasizer Nano ZS (Malvern Instrument Ltd., Worcestershire, UK). Each suspension was properly diluted before measurement to obtain suitable scattering intensity. ZP was measured using the same instrument to observe the particles’ electrophoretic mobility in the electric filed. Three replicates were taken for each sample. The displayed results are the average value ± *SD*.

#### Measurement of vesicular elasticity in terms of deformability index (DI)

Extrusion method was used for comparative assessment of the elasticity of the bilayer for the prepared elastosomes and DCN-loaded bilosomes (Van den Bergh et al., 2001). The vesicular dispersions were properly diluted (10 folds) before extrusion through 200 nm pore size nylon filter (Jinteng Experiment Equipment Co., Ltd, Tianjin, China) (El Zaafarany et al., 2010; Lei et al., 2013) under a constant pressure of 2.5 bar (Haug Kompressoren AG; Büchi Labortechnik AG, Flawil, Switzerland). The experiment was carried out as triplicate and the displayed results are the average value ± *SD*. DI was determined according to the following equation (Gupta et al., 2005):

(2) $$\text{DI}\;=\;J{\left(\frac{r_{\text{v}}}{r_{\text{p}}}\right)}^{2}$$

where *J* is the weight of dispersion extruded in 10 min, *r*_v_ is the size of vesicles after extrusion (nm), and *r*_p_ is the pore size of the barrier (nm).

#### *In-vitro* release of DCN elastosomes

A dialysis method was selected for determination of the release profiles of DCN from the prepared elastosomes (Khan et al., 2015). Cellulose dialysis membrane of molecular weight cutoff 12,000–14,000 Da (Spectrum Laboratories Inc., Rancho Dominguez, CA) was soaked in double-distilled water overnight before use for the experiment. An accurate amount of elastosomes vesicular dispersion, equivalent to 2.5 mg drug, was placed in the presoaked dialysis bag which was then clamped and placed in a beaker containing 50 mL of phosphate-buffered saline (PBS) pH 7.4, as a receptor compartment to simulate body physiological conditions (Auda et al., 2016; El-Say et al., 2016). The temperature was maintained at 32 °C with continuous stirring at 100 rpm using a magnetic stirrer (MSH-20 D Hotplate Stirrer Unit). At specified time intervals (0.5, 2, 4, 6, and 8 h), aliquots of 3 mL were withdrawn from receptor compartment and replaced by equal volume of fresh medium to maintain constant volume and preserve sink condition during the release study (Jain et al., 2013). Samples were then properly diluted and analyzed using HPLC system (Agilent Technologies, Santa Clara, CA) equipped with a P4000 pump unit and a Zorbax Extend C_18_ column (4.6 mm ×250 mm) containing 3.5 µm size adsorbent as stationary phase at *λ*_max_ of 254 nm. A mixture of acetonitrile and phosphate buffer in the ratio of 65:35 was used as mobile phase (pH 4) and delivered at a flow rate of 1 mL/min (Rao et al., 2009). The assay procedures were validated in terms of linearity, precision, and accuracy. The experiment was repeated 3 times and the results were expressed as average value ± *SD*.

### Studying the influence of the formulation variables using 4^1^.2^1^ full factorial design

A 4^1^.2^1^ full factorial design was employed for preparing DCN elastosomes using Design-Expert^®^ software (Stat-Ease, Inc., Minneapolis, MN). Two factors were evaluated in this design; X_1_: type of EA with four levels and X_2_: amount of EA with two levels. EE% (Y_1_), PS (Y_2_), PDI (Y_3_), ZP (Y_4_), and DI (Y_5_) were selected as dependent variables (Table 1). One-way analysis of variance (ANOVA) was performed to test the significance of each factor (*p* < .05) on the selected responses. For the type of EA (X_1_), post-hoc analysis was performed using Tukey’s honest significant difference (HSD) test using SPSS software 17.0 (SPSS Inc., Chicago, IL).

**Table 1.** The independent variables, their respective levels, and the model summary statistics of 4^1^.2^1^ full factorial design used for optimization of DCN elastosomes.

Factors (independent variables)	Levels of variables						
X_1_: type of EA X_2_: amount of EA (mg)	Brij S2 5		Brij C10		Cremophor EL	Cremophor RH 40 10	
Responses (dependent variables)	*R*^2^	Adjusted *R*^2^	Predicted *R*^2^	Constraints	*p* value	*F* value	Adequate precision
Y_1_: EE%	0.8109	0.6455	0.2438	Maximize	.0199	4.90	7.271
Y_2_: PS (nm)	0.9477	0.9019	0.7907	Minimize	.0002	20.70	12.468
Y_3_: PDI	0.5049	0.0718	−0.9802	Minimize	.4132	1.17	2.794
Y_4_: ZP (mV)	0.9644	0.9332	0.8576	Maximize (as absolute value)	<.0001	30.95	16.836
Y_5_: DI (g)	0.8309	0.6830	0.3236	Maximize	.0134	5.62	6.206

EE%: entrapment efficiency percentage; PS: particle size; PDI: polydispersity index; ZP: zeta potential; DI: deformability index.

### Optimization of DCN elastosomes

Design-Expert^®^ software was employed to select the optimal elastosomes formulation by applying the desirability function. The optimization process was planned to get a formula with the highest EE%, ZP (as absolute value), and DI and the least PS and PDI (Table 1). The solution with desirability value near to one was selected. For confirming the model efficacy, the selected formulation was prepared, characterized, and compared with the predicted responses. Furthermore, the characteristics of the optimal elastosomes formulation (EE%, PS, PDI, ZP, and DI) were compared with DCN-loaded bilosomes. The results were statistically analyzed by Student’s *t*-test using SPSS software 17.0.

### Transmission electron microscopy (TEM)

The morphology of the optimal elastosomes was visualized using TEM (Joel JEM 1230, Tokyo, Japan). A drop of the undiluted dispersion was stratified on a carbon coated copper grid and then left to dry at room temperature. Finally, the air dried sample was visualized at different magnifications at room temperature (25 °C) (El Zaafarany et al., 2010).

### Differential scanning calorimetry (DSC)

The thermal analysis of pure DCN, CH, Span 60, STC, EA, physical mixture of DCN with elastosomal components, and the optimal elastosomes was performed using differential scanning calorimetry (Shimadzu DSC-60, Shimadzu Corp., Kyoto, Japan) calibrated with purified indium (99.9%). Approximately 5 mg of each sample was mounted in standard aluminum pans and heated in a temperature range of 10–300 °C at a scanning rate of 10 °C/min under inert nitrogen flow (25 mL/min).

### *Ex-vivo* permeation studies

Newly born rats weighing 70 ± 20 g were killed and the skin was carefully excised from them. Subcutaneous tissues and adhering fats were removed by rubbing with cotton. The excised full thickness skin samples were equilibrated by soaking in PBS solution pH 7.4 at 4–8 °C about 1 h before beginning the experiment (Abdallah, 2013). Skin samples were then sandwiched securely between the donor and receptor compartments of a vertical Franz diffusion cell (1.76 cm^2^). SC was exposed to ambient condition (donor compartment) while the dermal side was batched with 50 mL of PBS pH 7.4 (receptor compartment) with temperature adjusted at 32 °C. The donor compartment was charged with 1 mL of one of the selected formulae, namely; the optimal elastosomes, DCN-loaded bilosomes and drug suspension (each containing 2.5 mg DCN) under non-occlusive condition. At predetermined time intervals (0.5, 1, 2, 4, 6, 8, and 24 h), samples from the receptor fluid (3 mL) were withdrawn and the cell was refilled by equal volume of freshly prepared receptor fluid. The experiment was conducted in triplicate and the results were calculated as mean ± *SD*. The withdrawn samples were then analyzed using a validated HPLC method as previously discussed. The cumulative amount of drug permeated through the skin per unit area (µg/cm^2^) was plotted against time (h) (Abdallah 2013). The flux (*J*_max_) at 24 h and the enhancement ratio (ER) were calculated according to the following equations (El Zaafarany et al., 2010):

(3) $$J_{\text{max}}\;\text{=}\;\frac{\text{amount}\;\text{of}\;\text{drug}\;\text{permeated}}{\text{time}\;\text{×}\;\text{area}\;\text{of}\;\text{membrane}}$$

(4) $$\text{ER}\;\text{=}\;\frac{J_{\text{max}}\;\text{of}\;\text{the}\;\text{nanovesicles}}{J_{\text{max}}\;\text{of}\;\text{the}\;\text{drug}\;\text{suspension}\;\text{(control)}}$$

Flux values and amount of DCN permeated from each vesicle and drug suspension were analyzed by one-way ANOVA using SPSS software 17.0. Post-hoc analysis was performed using Tukey’s HSD test. Difference at *p* ≤ .05 was considered significant.

### *In-vivo* studies

The protocol of the studies (PI 1738) was evaluated and approved by the Research Ethics Committee in the Faculty of Pharmacy, Cairo University, Egypt. The use and the treatment of animals in all studies were conformed to the EU Directive 2010/63/EU for animal experiments (Tadros & Al-Mahallawi, 2015).

#### *In-vivo* skin deposition studies

A total of 72 male Wistar rats, weighing 150–200 g, were involved in the study. The animals were supplied with standard diet and tap water *ad libitum*. On the experiment day, the rats were randomly separated into 4 groups with 18 animals in each group, where group I behaved as control, whereas animals in groups II, III, and IV received topical application of the optimal elastosomes, DCN-loaded bilosomes, and drug suspension, respectively. Bottle caps that served as drug pools (4.15 cm^2^) were stuck to rat’s dorsal skin which was shaved to remove hair with an electric clipper 24 h before application of the sample (Shen et al., 2014). Half milliliter of each formulation was added non-occlusively into the drug pool. After different time intervals of treatments’ application (1, 2, 4, 6, 8, and 10 h), three animals from each group were killed using an overdose of anesthetic ether and the dorsal rat skin that was in contact with the formulation was excised then immediately washed with 10 mL of normal saline in two divided portions. The excised skin sections were cut into pieces and sonicated in 5 mL dimethyl sulfoxide (DMSO) for 30 min. The skin homogenate was then filtered through a 0.22 µm filter membrane and the concentration of DCN was determined using a validated HPLC method as previously mentioned. The skin deposition of DCN was calculated from the obtained data. Statistical significance was analyzed by one-way ANOVA using SPSS software 17.0. Post-hoc analysis was performed using Tukey’s HSD test. Difference at *p* ≤ .05 was considered significant. The destruction of animal carcasses was achieved by incineration at the end of experiment.

#### *In-vivo* histopathological study

The study was conducted using eight male Wistar rats, weighing 150–200 g, that were randomly divided into 2 groups with 4 animals each. Group I acted as control, whereas animals in group II received topical application of the optimal elastosomes onto the skin surface 3 times daily for a period of 1 week (Abdelbary & AbouGhaly, 2015). The animals were then killed and the skin was excised for histopathological examination according to the procedures reported by Bancroft & Gamble (2008). Briefly, the skin samples were mounted in 10% formal saline for 24 h then washed with tap water and then serial dilutions of alcohols were used for dehydration. Specimens were then cleared in xylene and embedded in paraffin beeswax blocks and kept at 56 °C for 24 h. Sections from the paraffin blocks of 4 mm thickness were cut using a microtome (Leica Microsystems SM2400, Cambridge, UK), collected on glass slides, deparaffinized, stained with hematoxylin and eosin, and then observed under an electric light microscope.

#### *In-vivo* pharmacokinetic study

##### Study design

Six male albino rabbits (2–2.5 kg) were involved in the study. The rabbits were housed individually in cages under standard laboratory conditions. On the experiment day, rabbits were divided into two groups of equal number. A simple randomized crossover design was conducted on two phases with wash-out period of 1 week. In phase I, the hair on the dorsal side of one group was removed using electric clipper and the optimal elastosomes (equivalent to 25 mg DCN) was applied over a specified area (10 cm ×5 cm). On the other hand, an equivalent DCN suspension (25 mg DCN) was orally administered to the other group using a plastic syringe. In the second phase, reverse randomization took place. At different time intervals (1, 2, 3, 4, 6, 8, 12, and 24 h) following application, blood samples (3 mL) were withdrawn from the marginal ear vein then transferred to tubes containing heparin to prevent blood coagulation. Plasma was then separated by centrifugation at 4000 rpm for 15 min and stored at −20 °C till assayed.

##### Assay method

All frozen human plasma samples were thawed at ambient temperature. Rhein (the active metabolite of DCN) was analyzed in plasma samples using a sensitive, accurate, and selective liquid chromatography-mass spectrometry (LC/MS/MS). Half mL plasma samples were placed in 5 mL glass tubes and then 100 µL of hydrochlorothiazide (HCTZ) as internal standard (IS, 100 ng/mL) solution in acetonitrile was added and the samples were vortexed. In addition, acetonitrile (1 mL) was added to samples followed by vortexing for 2 min. The tubes were then centrifuged at 4000 rpm for 10 min at 4 °C (Eppendorf centrifuge 5804 R, Hamburg, Germany). The upper organic layer was transferred into the auto sampler vials of the LC/MS/MS. A Shimadzu Prominence (Shimadzu Scientific Instruments, Columbia, MD) series LC system equipped with degasser (DGU-20A3) and solvent delivery unit (LC-20AB) along with an autosampler (SIL-20 AC) was employed to inject samples of 20 µL on Luna C_18_ column (50 × 4.6 mm) containing 5 μm PS adsorbent as stationary phase (Phenomenex Inc., Torrance, CA). The guard column was a Phenomenex C18 (5 × 4.0 mm) of 5 μm PS. The isocratic mobile phase consisted of acetonitrile and 0.02 M ammonium acetate buffer (7:3 v/v) in addition to 0.1% formic acid and was delivered at a flow rate of 0.5 mL/min into the mass spectrometer’s electrospray ionization chamber. Quantitation was achieved by MS/MS detection in the negative ion mode using a MDS Sciex (Foster City, CA) API-3200 mass spectrometer, equipped with a turbo ion spray interface at 450 °C. The ion spray voltage was set at −4500 V. The common parameters: curtain gas, nebulizer gas, collision gas, and auxiliary gas were set at 20 psi, 30 psi, 6 psi, and 40 psi, respectively. The compound parameters: declustering potential, collision energy, entrance potential, and collision exit potential were −45 V, −22 V, −10 V, and −12 V for rhein and −90 V, −26 V, −10 V, and −23 V for HCTZ (IS), respectively. Detection of the ions was performed in multiple reaction monitoring mode, monitoring the transition of the *m/z* 282.86 precursor ion to the *m/z* 238.90 for rhein and *m/z* 295.69 precursor ion to the *m/z* 268.90 for IS. Quadrupoles Q1 and Q3 were set on unit resolution. The analytical data were processed by Analyst^®^ Software Version 1.6 (AB Sciex Pte. Ltd., Woodlands, Singapore).

##### Pharmacokinetic parameters and statistical analysis

The mean rhein plasma concentrations were plotted against time. Plasma concentration-time data of rhein was analyzed for each rabbit by non-compartmental pharmacokinetic models using computer program (Kinetica^®^ 5, Thermo Fisher Scientific Inc., Waltham, MA). The peak plasma concentration (*C*_max_) and time for reaching this peak (*T*_max_) were obtained from the individual plasma concentration-time curves. Area under concentration-time curve till last quantifiable point (AUC_0→24_) was calculated by the linear trapezoidal rule. Area under concentration-time curve from time zero till infinity (AUC_0→∞_) was calculated as AUC_0→∞_ = AUC_0→24_ + C_t_/K, where C_t_ is the last measured concentration at the time t. The pharmacokinetic parameters (*C*_max_, AUC_0→24_, and AUC_0→∞_) were compared between both treatments by Student’ s *t*-test using SPSS software 17.0. The nonparametric Signed Rank Test (Mann–Whitney’s *U* test) was used to compare the medians of *T*_max_ for both treatments using the same software. Difference at *p* ≤ .05 was considered significant.

#### *In-vitro/in-vivo* correlation

For the purpose of IVIVC, DCN oral suspension was subjected to *in-vitro* release study by the previously mentioned manner that was used for assessment of the release of DCN elastosomes. The release medium was kept at 37 ± 0.5 °C to simulate the physiological environment of gastrointestinal tract (Jain et al., 2013). The correlation between the *in-vitro* release and *in-vivo* drug absorption was investigated for both the optimal elastosomes and DCN oral suspension using multiple level C IVIVC. The partial AUCs (AUC_0→2_, AUC_0→4_, and AUC_0→6_), calculated using the linear trapezoidal rule, were correlated with the cumulative percentage DCN released obtained from the *in-vitro* release profiles at three time points (2, 4, and 6 h). *In-vitro* and *in-vivo* data were displayed as independent (X) and dependent (Y) variables, respectively. The correlation coefficient (R) and the slope of each line (b) were calculated by linear regression analysis using Microsoft Excel 2010 (Microsoft corporation, Washington, DC) (Lake et al., 1999; Volpato et al., 2004).

## Results and discussion

### Factorial design analysis

The used design was a 4^1^.2^1^ full factorial design which was statistically analyzed using Design-Expert^®^ software. Each factor’s levels were set on the basis of preliminary experiments and feasibility of preparing elastosomes at these values. The model selected was two-factor interaction (2 FI). Adequate precision measured the signal to noise ratio to make sure that the model can be used to navigate the design space (De Lima et al., 2011). A ratio greater than 4 is desirable which was observed in all responses except PDI as shown in Table 1. The adjusted and predicted *R*^2^ values are preferred to be close to each other in order to be in reasonable agreement (DeLoach & Ulbrich, 2007) which was achieved in all responses except PDI (Table 1). The negative predicted *R*^2^ value of PDI implies that the overall mean is a better predictor of the response. This might be attributed to that PDI was not affected by the studied factors.

#### Effect of formulation variables on EE% of DCN elastosomes

**Figure 1.**
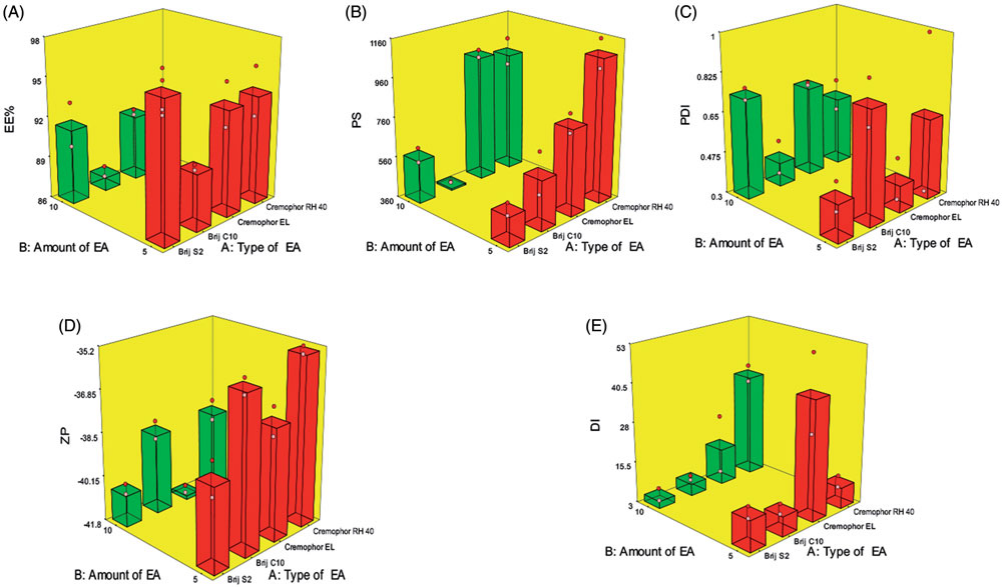
Response 3D plots for the effect of type of EA (X_1_) and amount of EA (X_2_) on (A) entrapment efficiency percentage (EE%), (B) particle size (PS), (C) polydispersity index (PDI), (D) zeta potential (ZP), and (E) deformability index (DI) of DCN elastosomes.

The percentage of DCN entrapped within the elastosomes ranged from 87.00 ± 0.56 to 96.25 ± 2.19% (Table 2). The influence of type of EA (X_1_) and amount of EA (X_2_) on EE% of DCN elastosomes is illustrated graphically as response 3D plot in Figure 1(A). The resulting equation in terms of coded factors was as follows:

$$\text{EE}\%\;=+\text{ 91}.\text{91}+\text{1}.{\text{99X}}_{\text{1}}-\text{ 3}.{\text{31X}}_{1}^{2}+\;0.{\text{46X}}_{1}^{3}-\text{ 1}.{\text{68X}}_{\text{2}}-\;0.{\text{67X}}_{\text{1}}{\text{X}}_{\text{2}}+\;0.0{\text{76X}}_{1}^{2}{\text{X}}_{\text{2}}+\;0.\text{2}0{\text{X}}_{1}^{3}{\text{X}}_{\text{2}}.$$

**Table 2.** Experimental runs, independent variables, and measured responses of the 4^1^.2^1^ full factorial experimental design of DCN elastosomes compared to DCN-loaded bilosomes (A) and the observed and predicted values of the optimal elastosomes (E1) (B).

	X_1_: type of EA	X_2_: amount of EA (mg)	Y_1_ EE%^a^	Y_2_ PS (nm)^a^	Y_3_ PDI^a^	Y_4_ ZP (mV)^a^	Y_5_ DI (g)^a^
(A) Formulations							
E1	Brij S2	5	96.25 ± 2.19	506.35 ± 44.61	0.46 ± 0.09	−38.65 ± 0.91	12.74 ± 2.63
E2	Brij S2	10	91.50 ± 2.33	576.65 ± 53.38	0.74 ± 0.04	−40.60 ± 0.28	5.25 ± 3.15
E3	Brij C10	5	90.20 ± 0.28	611.50 ± 150.61	0.79 ± 0.14	−35.90 ± 0.42	9.28 ± 2.29
E4	Brij C10	10	87.00 ± 0.56	368.75 ± 3.46	0.40 ± 0.11	−38.75 ± 0.71	6.75 ± 1.06
E5	Cremophore EL	5	93.85 ± 2.33	796.75 ± 69.22	0.41 ± 0.13	−37.55 ± 3.39	40.75 ± 17.30
E6	Cremophor EL	10	90.89 ± 0.27	1000.95 ± 125.93	0.69 ± 0.01	−41.65 ± 0.21	14.50 ± 13.43
E7	Cremophor RH 40	5	94.05 ± 2.61	1087.00 ± 103.23	0.27 ± 0.09	−38.35 ± 0.21	9.65 ± 2.61
E8	Cremophor RH 40	10	91.50 ± 1.69	971.50 ± 96.87	0.60 ± 0.10	−38.80 ± 0.56	35.47 ± 3.78
DCN-loaded bilosomes	–	–	100.00 ± 0.00	301.65 ± 17.32	0.39 ± 0.01	−40.15 ± 3.32	7.77 ± 0.92
(B) E1 (optimal elastosomes)							
Observed values			96.25	506.35	0.46	−38.65	12.74
Predicted values			97.20	498.73	0.54	−39.34	14.33

EE%: entrapment efficiency percentage; PS: particle size; PDI: polydispersity index; ZP: zeta potential; DI: deformability index. All the prepared vesicles contained equal amounts of DCN, Span 60, STC, and CH.

a Data presented as mean ± *SD* (*n* = 3).

Type of EA (X_1_) had a significant effect on EE% (*p* = .0152). Brij C10, the relatively shortest alkyl chain length surfactant, was shown to form vesicles with the lowest EE% due to the direct relationship between the alkyl chain length and EE% (Hao et al., 2002). Increasing the alkyl chain length of surfactant increases the EE% for hydrophobic drugs, like DCN, by increasing the hydrophobic area within the vesicular bilayer (Duangjit et al., 2014). Moreover, by increasing the alkyl chain length, stronger van der Waals interactions would be formed between the adjacent lipid chains which requires more energy for disrupting this ordered packing and releasing the entrapped drug (Mohammed et al., 2004). Increasing amount of EA (X_2_) significantly decreased EE% (*p* = .0058) by either destabilization of the vesicular bilayer with resultant pore formation or by increasing drug solubilization and diffusion into the aqueous media during vesicular preparation (Salama et al., 2012; Al-Mahallawi et al., 2014).

#### Effect of formulation variables on PS of DCN elastosomes

Developing vesicles with optimum PS was one of our most important intentions for enhancing the transdermal flux of DCN. PS of the prepared elastosomes fluctuated from 368.75 ± 3.46 to 1087.00 ± 103.23 nm (Table 2). The influence of type of EA (X_1_) and amount of EA (X_2_) on PS of DCN elastosomes is illustrated graphically as response 3D plot in Figure 1(B). The resulting equation in terms of coded factors was as follows:

$$\text{PS }=+\text{ 739}.\text{93 }-\text{ 198}.{\text{43X}}_{\text{1}}-\text{ 249}.{\text{81X}}_{\text{1}}{}^{\text{2}}+\text{158}.{\text{92X}}_{\text{1}}{}^{\text{3}}-\text{ 1}0.{\text{47X}}_{\text{2}}+\text{ 45}.{\text{62X}}_{\text{1}}{\text{X}}_{\text{2}}-\text{ 11}0.{\text{91X}}_{\text{1}}{{}^{\text{2}}}_{}{\text{X}}_{\text{2}}+\text{ 112}.{\text{57X}}_{\text{1}}{}^{\text{3}}{\text{X}}_{\text{2}}.$$

Only type of EA (X_1_) significantly influenced PS of the prepared vesicles (*p* < .0001). Cremophors formed significantly larger vesicles compared to Brij-derived elastosomes. This could be attributed to that Cremophor EL and Cremophor RH 40 possess 35 and 40 hydrophilic polyethylene oxide (PEO) units, respectively compared to two units for Brij S2 and 10 units for Brij C10. Hence, Cremophors are more hydrophilic which resulted in increasing the water uptake by the vesicles with resultant increase in PS (Abdelbary & Aburahma, 2015).

#### Effect of formulation variables on PDI of DCN elastosomes

PDI of elastosomal formulae ranged from 0.27 ± 0.09 to 0.79 ± 0.14 (Table 2). The influence of type of EA (X_1_) and amount of EA (X_2_) on PDI of DCN elastosomes is illustrated graphically as response 3D plot in Figure 1(C). It was revealed that PDI of the prepared elastosomes was not significantly affected by any of the studied factors. The resulting equation in terms of coded factors was as follows:

$$\text{PDI}=\;+\;0.\text{6 }+\text{ 5}.000\text{E}‐00{\text{3X}}_{\text{1}}+\text{ 3}.000\text{E}‐00{\text{3X}}_{\text{1}}{}^{\text{2}}-\;0.0{\text{39X}}_{\text{1}}{}^{\text{3}}+\;0.0{\text{14X}}_{\text{2}}+\;0.{\text{12X}}_{\text{1}}{\text{X}}_{\text{2}}-0.{\text{21X}}_{\text{1}}{{}^{\text{2}}}_{}{\text{X}}_{\text{2}}+\;0.{\text{12X}}_{\text{1}}{}^{\text{3}}{\text{X}}_{\text{2}}.$$

#### Effect of formulation variables on ZP of DCN elastosomes

All DCN elastosomes obtained negative ZP values which ranged from −35.90 ± 0.42 to −41.65 ± 0.21 mV (Table 2). Since all formulations obtained negative ZP values, absolute values are used for discussion to prevent misperception. The influence of type of EA (X_1_) and amount of EA (X_2_) on ZP of DCN elastosomes is illustrated graphically as response 3D plot in Figure 1(D). The resulting equation in terms of coded factors was as follows:

$$\text{ZP }=-\text{38}.\text{41}-\text{1}.{\text{22X}}_{\text{1}}+\text{1}.0{\text{8X}}_{\text{1}}{}^{\text{2}}-\text{ 1}.{\text{19X}}_{\text{1}}{}^{\text{3}}-\text{ 1}.{\text{54X}}_{\text{2}}+\;0.{\text{57X}}_{\text{1}}{\text{X}}_{\text{2}}+\;0.{\text{12X}}_{\text{1}}{{}^{\text{2}}}_{}{\text{X}}_{\text{2}}-\;0.{\text{51X}}_{\text{1}}{}^{\text{3}}{\text{X}}_{\text{2}}.$$

Type of EA (X_1_) was found to significantly affect ZP of the prepared elastosomes (*p* = .0002). Regarding Brij surfactants, Brij C10 formed vesicles with significantly lower ZP values compared to Brij S2. On the other hand, Cremophor RH 40 based vesicles obtained significantly lower ZP values than the vesicles containing Cremophor EL. This would be attributed to that EAs, based on their hydrophilicty, could reside on the vesicles’ surface leading to masking of their charge with resultant lower ZP values (Wilson et al., 2008). Accordingly, Brij C10 (being more hydrophilic (HLB 12) compared to Brij S2 (HLB 4)) could cause more shielding of the negative surface charge by residing on the vesicular bilayer surface resulting in significantly lower ZP values. Similarly, the lower ZP values of Cremophor RH 40 vesicles compared to Cremophor EL ones could be attributed to the higher hydrophilicity of Cremophor RH 40 (HLB 16) than Cremophor EL (HLB 13). Furthermore, increasing amount of EA (X_2_) significantly increased ZP values (*p* < .0001). This might be attributed to the decreased EE% when the amount of EA increased as previously discussed. DCN, being an acidic drug with ionizable carboxylic group, could ionize and acquire negative charge in the neutral and alkaline pH. Hence, by increasing amount of EA, more unentrapped ionizable DCN would be available and hence contributes to increasing the charge density of the dispersion.

#### Effect of formulation variables on DI of DCN elastosomes

The degree of deformability of vesicles is a crucial parameter for transdermal drug delivery to facilitate their squeezing through the minute skin pores (Kakkar & Kaur, 2011). The deformability of the prepared elastosomes is presented in terms of DI which ranged from 5.25 ± 3.15 to 40.75 ± 17.30 g (Table 2). The effect of type of EA (X_1_) and amount of EA (X_2_) on the DI of DCN elastosomes is illustrated graphically as response 3D plot in Figure 1(E). The resulting equation in terms of coded factors was as follows:

$$\text{DI}=\;+\text{16}.\text{8}0\;-\text{ 7}.\text{8}0{\text{X}}_{\text{1}}-\text{ 8}.{\text{78X}}_{\text{1}}{}^{\text{2}}+\text{1}0.{\text{83X}}_{\text{1}}{}^{\text{3}}-\text{ 1}.{\text{31X}}_{\text{2}}-\text{ 2}.{\text{44X}}_{\text{1}}{\text{X}}_{\text{2}}+\;0.0{\text{41X}}_{\text{1}}{{}^{\text{2}}}_{}{\text{X}}_{\text{2}}-\text{11}.{\text{82X}}_{\text{1}}{}^{\text{3}}{\text{X}}_{\text{2}}.$$

Only type of EA (X_1_) had a significant impact on DI (*p* = .0202). Cremophor-derived vesicles were shown to be more deformable than those containing Brij surfactants. As previously mentioned, Brij surfactants are more hydrophobic (lower HLB) than Cremophors (higher HLB) which may result in reducing the formation of the transient hydrophilic holes within the bilayer and consequently decreases membrane fluidity (El Zaafarany et al., 2010).

### *In-vitro* release of DCN elastosomes

**Figure 2.**
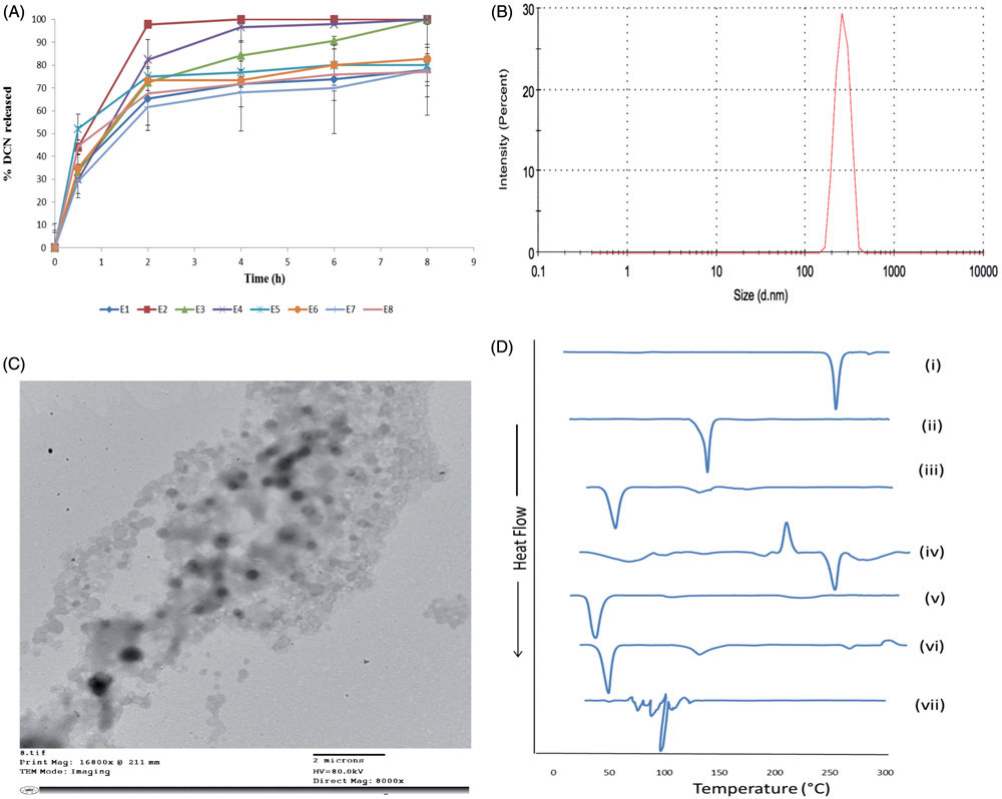
The release profiles of DCN from the prepared eight elastosomal formulations (A). The size distribution curve (B), transmission electron micrograph (C) of E1 and DSC thermograms of (i) DCN, (ii) CH, (iii) Span 60, (iv) STC, (v) Brij S2, (vi) physical mixture of DCN with elastosomal components and (vii) E1 (D).

Figure 2(A) represents the release profiles of the prepared DCN elastosmal formulae. It was obvious that DCN was released from the vesicles in a slow manner up to 8 h. Hence, elastosomes could retard the drug transfer across its bilayered compartment due to the presence of CH in equal amount in all formulae which prevented drug leakage from the vesicular formulation by abolishing gel to liquid transition of the nanovesicles (Tamizharasi et al., 2009).

### Selection of the optimal formulation

The optimization of pharmaceutical formulations is aiming at tailoring the independent formulation variables to produce a high-quality product with the optimum physico-chemical properties (Al-Mahallawi et al., 2014). Thus, the desirability function was applied to select the optimal formulation from the prepared 8 formulae according to the full factorial design. The desirability constraints for the optimal formulation (maximizing EE%, ZP (as absolute value), and DI and minimizing PS and PDI) were established in E1with overall desirability 0.720. E1 was prepared using 5 mg of Brij S2 and showed EE% of 96.25 ± 2.19%, PS of 506.35 ± 44.61 nm, PDI of 0.46 ± 0.09, ZP of −38.65 ± 0.91 mV, and DI of 12.74 ± 2.63 g. The size distribution curve of E1 is shown in Figure 2(B). As shown in Table 2, there was a high similarity between the observed and predicted values of E1. No significant difference was observed (*p* > .05). This confirmed the reasonableness of the optimization process and the suitability of using E1 for further investigation. Furthermore, E1 showed significantly higher DI value than DCN-loaded bilosomes (*p* < .05) which confirmed the impact of EAs on enhancing the vesicular deformability and skin permeation properties (Kakkar & Kaur, 2011). Oppositely, there was no significant difference in EE%, PS, PDI, and ZP between both vesicles (Table 2).

### Transmission electron microscopy (TEM)

E1 showed spherical non-aggregating vesicles with smooth surface and sharp boundaries as illustrated in Figure 2(C). Furthermore, the diameter of E1 observed by TEM micrographs was in a good agreement with the size obtained by the Zetasizer.

### Differential scanning calorimetry (DSC)

Figure 2(D) represents the thermograms of pure DCN, CH, Span 60, STC, Brij S2, physical mixture of DCN with elastosomal components and E1. The DSC scan of pure DCN depicted a single endothermic peak at 256.19 °C due to its crystalline nature (Elsayed et al., 2014). The thermogram of CH depicted sharp endothermic transition at 146.81 °C because of degradation (Rudra et al., 2010). Span 60 and Brij S2 showed endothermic peaks at 55.01 and 49.88 °C, respectively, corresponding to their melting points. STC showed exothermic crystallization peak at 238.30 °C followed by endothermic one at 264.75 °C due to melting and hydration of the hydrophilic head in STC structure (Trivedi et al., 2015). With respect to the physical mixture of DCN with elastosomal components, the endothermic transition of DCN was shown with significant lower intensity compared to pure drug due to its dilution with the used excipients (Elsayed et al., 2014). Oppositely, complete absence of DCN peak was observed in thermogram of E1 confirming that DCN was entrapped in elastosomes with good interaction with their vesicular bilayer. Furthermore, the complete disappearance of characteristic peaks of STC ensured its fluidization effect on the vesicular bilayers (Al-Mahallawi et al., 2015).

### *Ex-vivo* skin permeation study

**Figure 3.**
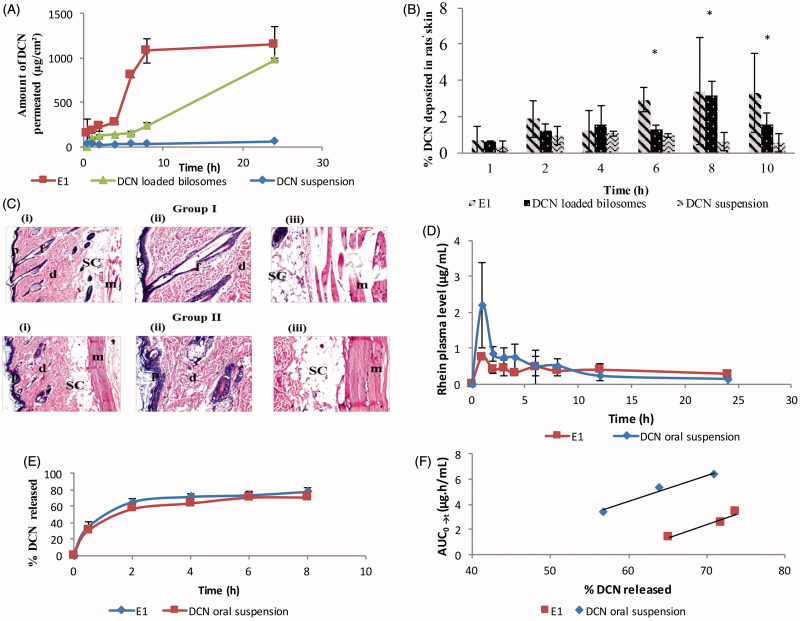
*Ex-vivo* permeation profiles (A), *in-vivo* skin deposition plot (B) of DCN from E1, DCN-loaded bilosomes and DCN suspension after topical application (*p < .05). Photomicrographs showing histopathological sections (stained using hematoxylin and eosin) of normal untreated rat skin (group I) and rat skin treated with E1 (group II) (C). (i)–(iii) denote the magnification power of 16× to illustrate all skin layers, 40× to identify the epidermis and dermis, and 40× to identify the subcutaneous tissue and muscles, respectively. Abbreviations: epidermis (p), dermis (d), hair follicles (f), subcutaneous tissue (sc), and muscle (m). Mean rhein plasma-concentration time curves (D), release profiles (E), and multiple level C IVIVC between AUC_0→_*_t_* for 2, 4, and 6 h and percentage DCN released at these time points (*b* = 0.22; *R* = 0.976 and *b* = 0.21; *R* = 0.984 for E1 and DCN oral suspension, respectively) (F) for E1 and DCN oral suspension.

The permeability of DCN from E1 was investigated and its permeation profile was compared to that obtained from DCN-loaded bilosomes and drug suspension as shown in Figure 3(A). Comparing the skin permeability parameters, both E1 and DCN-loaded bilosomes showed significantly increased drug flux and resulted in higher amount permeated per unit area in 24 h than drug suspension (*p* < .05) (Table 3). The ER was more than 19 times for E1 and more than 16 times for DCN-loaded bilosomes compared to drug suspension. Furthermore, E1 showed numerically (but not significantly) higher skin permeability parameters than DCN-loaded bilosomes (Table 3). This significant enhancement in the transdermal DCN delivery from the prepared nanovesicles was attributed to the ability of the vesicular carriers to introduce DCN as fine dispersion compared to the coarser PS of the drug suspension which increased the surface area and consequently decreased the diffusional path length of DCN through the skin (Fahmy, 2015). Besides, the presence of EAs in elastosomal constructs was behind its enhanced skin permeation potential. EAs could enhance the vesicular deformability and consequently increase its affinity to retain and bind water when applied under non-occlusive condition to avoid dehydration (Walve et al., 2011). Hence, it migrates deeply to water rich strata carrying drug molecules to secure adequate hydration condition. Furthermore, EAs have great propensity for highly curved structure that allows vesicles to undergo stress-dependent adjustment of their local composition to overcome resistance of their motion through the confining channel of the skin and permit drug transport reproducibly and noninvasively (Cevc & Blume, 2003).

**Table 3.** Skin permeability parameters of DCN after topical application of E1, DCN-loaded bilosomes, and drug suspension (A) and pharmacokinetic parameters of E1 in comparison with DCN oral suspension (B).

(A)		Treatment	
Skin permeability parameters	E1	DCN-loaded bilosomes	Drug suspension
Total amount of drug permeated per unit area in 24 h (µg/cm^2^)^a^	1150.92 ± 194.71	1003.50 ± 47.38	59.44 ± 0.50
*J*_max_ (µg/cm^2^/h)^a^	27.24 ± 4.61	23.75 ± 1.12	1.41 ± 0.01
ER	19.36	16.84	1
(B)			
*In-vivo* pharmacokinetic parameters	E1		DCN oral suspension
*C*_max_ (µg/mL)^b^	0.76 ± 0.04		2.24 ± 1.12
*T*_max_ (h)^c^	1		1
AUC_0→24_ (µg h/mL)^b^	9.26 ± 3.02		9.95 ± 1.04
AUC_0→∞_ (µg h/mL)^b^	20.65 ± 16.40		15.49 ± 10.04

*J*_max_: flux; ER: enhancement ratio.

a Data presented as mean ± *SD* (*n* = 3).

b Data presented as mean ± *SD* (*n* = 6).

c Data presented as median.

### *In-vivo* studies

#### *In-vivo* skin deposition study

Skin deposition of DCN from E1, DCN-loaded bilosomes, and drug suspension is shown in Figure 3(B). It was obvious that DCN showed significantly higher skin deposition from the prepared vesicles than drug suspension (*p* < .05) at 8 h and 10 h. The percentage of DCN deposited from E1 and DCN-loaded bilosomes at the end of study period was 5.87 and 2.82 times, respectively, compared to drug suspension. Furthermore, the percentage of DCN deposited in rats’ skin at 6 h was significantly higher for E1 compared to DCN-loaded bilosomes and drug suspension (*p* < .05). The higher skin deposition of DCN from the prepared vesicles was due to the ability of bile salt in their constructs to form chemical compounds with keratocytes via interaction of the anionic head group with the cationic sites of skin proteins (Som et al., 2012). Hence, vesicles can act as drug carriers which penetrate deeply to the target site of dermis where a depot, from which the drug can be released, is formed (Ma et al., 2015). Moreover, the significantly higher skin deposition of DCN from E1 at 6 h was due to the ability of elastosomes, being highly deformable vesicles, to partition themselves through SC interstices carrying the entrapped DCN to deeper tissues (Peira et al., 2007).

#### *In-vivo* histopathological study

Figure 3(C) demonstrates the light microscopy examination of stained rat skin sections obtained from group I (untreated control) and group II (skin treated with E1). The untreated control showed normal skin architecture with well-defined epidermis, dermis, subcutaneous tissue, and muscles. The epidermis revealed stratified squamous keratinized epithelium that was supported by a dermis layer of dense fibroelastic connective tissue that was devoid of any inflammatory cells. With respect to group II, there was no histopathological alteration and the normal histopathological structure of the epidermal layer as well as the underlying dermis with sebaceous glands and hair follicles was observed. This confirms that DCN elastosomes showed acceptable safety profile when applied topically.

#### *In-vivo* pharmacokinetic study

The rhein mean plasma-concentration time curves after topical application of E1 and oral administration of DCN suspension are shown in Figure 3(D). The mean pharmacokinetic parameters namely; *C*_max_, *T*_max_, AUC_0→24_, and AUC_0→∞_ are presented in Table 3. Statistical analysis showed that there was no significant difference between E1 and DCN oral suspension in aspects of *C*_max_, *T*_max_, AUC_0→24_, and AUC_0→∞_ with *p* values of .17, .317, .541, and .485, respectively. The aforementioned results confirmed that elastosomes could act as effective transdermal delivery system of DCN in order to avoid its oral gastrointestinal side effects without significantly altering the rate and extent of absorption.

#### *In-vitro/in-vivo* correlation

There are different levels of IVIVC as defined in the literature (level A, level B, level C, and multiple level C). Multiple level C correlates one or more pharmacokinetic parameters to the percentage of drug released at different time points of the release profile. This level of correlation could be considered as useful as level A IVIVC (Volpato et al., 2004). Therefore, it was selected in this study for developing correlation between the *in-vitro* and *in-vivo* reports of both E1 and DCN oral suspension. The release profiles of both E1 and DCN oral suspension are shown in Figure 3(E). As shown in Figure 3(F), reasonable IVIVCs were observed for both E1 and DCN oral suspension when percentage DCN released was correlated with the selected pharmacokinetic parameter (AUC) at different time points. The slopes as well as correlation coefficient values for both lines were similar (*b* = 0.22; *R* = 0.976 and *b* = 0.21; *R* = 0.984 for E1 and DCN oral suspension, respectively). This observed high similarity between the two plots supported the results of *in-vivo* pharmacokinetic study that E1 showed no significant difference in aspects of rate and extent of DCN absorption compared to oral suspension.

## Conclusions

In this work, we formulated elastosomes as a novel transdermal delivery system of DCN. Eight elastosomal formulations were prepared using film hydration technique according to a 4^1^.2^1^ full factorial design which was used to select the optimal elastosomes (E1) which showed promising skin permeation potential and retention capacity due to its ultra-deformable properties. Moreover, the *in-vivo* histopathological study ensured the safety and non-irritancy of E1 when applied on rats’ skin. Additionally, E1 showed comparable pharmacokinetic parameters for DCN as that obtained after oral administration of DCN suspension. Multiple level C IVIVC showed that both E1 and DCN oral suspension displayed a good correlation between their *in-vitro* release data and *in-vivo* drug performance. Concisely, the results confirmed that elastosomes can be considered as a promising solution for boosting DCN transdermal delivery and avoiding the oral problems of DCN.
